# Association of Pelvic Inflammatory Disease with Risk of Endometriosis: A Nationwide Cohort Study Involving 141,460 Individuals

**DOI:** 10.3390/jcm7110379

**Published:** 2018-10-24

**Authors:** Fei-Wu Tai, Cherry Yin-Yi Chang, Jen-Huai Chiang, Wu-Chou Lin, Lei Wan

**Affiliations:** 1 School of Chinese Medicine, China Medical University, Taichung 40402, Taiwan; u102022306@cmu.edu.tw; 2 Department of Obstetrics and Gynecology, China Medical University Hospital, Taichung 40447, Taiwan; changyinyi@hotmail.com; 3 School of Medicine, China Medical University, Taichung 40402, Taiwan; 4 Management Office for Health Data, China Medical University Hospital, Taichung 40447, Taiwan; zinvii@gmail.com; 5 Department of Biotechnology, Asia University, Taichung 41354, Taiwan

**Keywords:** pelvic inflammatory disease, endometriosis, inflammation, reproductive tract microbiota, upper genital tract infection

## Abstract

Endometriosis occurs when endometrial tissue exists outside the uterine cavity. The presence of ectopic endometrial tissue and resultant inflammation cause serious symptoms, including chronic pelvic pain, dysmenorrhea, dyspareunia, and infertility. Pelvic inflammatory disease is caused by the ascension of pathogenic bacteria from the vagina to the uterus, Fallopian tubes, and ovaries. The factors involved in the pathogenesis of the two conditions are not clearly understood, but recent studies have suggested that disturbances of the female reproductive tract microbiota and inflammatory processes influence the development of both diseases. Using data from the National Health Insurance Research Database (NHIRD), we conducted a study to assess the association of pelvic inflammatory disease (PID) with endometriosis. An age-matched control group including patients without PID was selected. Patients with a pre-existing diagnosis of endometriosis were excluded. This nationwide retrospective cohort study, involving a total of 141,460 patients, demonstrated that patients with PID had a three-fold increase in the risk of developing endometriosis (HR = 3.02, 95% CI = 2.85–3.2).

## 1. Introduction

Endometriosis is a condition in which endometrial epithelial and stromal tissue exist outside the uterine cavity [1]. Although the disease itself is not a malignant process, it can cause debilitating symptoms, including dysmenorrhea, dyspareunia, chronic pain, and infertility. The growth and cyclical bleeding of endometrial implants and resultant inflammation increase the production of pro-inflammatory cytokines. These processes eventually lead to the development of pelvic adhesions and endometriomas, which distort anatomical structure and interfere with reproductive function. [2]

The mechanisms involved in the pathogenesis of endometriosis remain a mystery. At present, the retrograde menstruation theory remains the most widely embraced: endometrial cells shed during menstruation flow backward through the Fallopian tubes and are “regurgitated” into the peritoneal cavity, where they implant and develop into ectopic lesions [3]. It is important to note that although retrograde menstruation occurs in up to 90% of women, only 6–10% of all menstruating women develop endometriosis [1,4]. This suggests that other factors contribute to the establishment and survival of ectopic endometriotic implants. 

Previous studies have shown that inflammatory cytokines such as interleukin-1 beta, interleukin-6, and tumor necrosis factor promote the implantation of ectopic endometrial tissue [1,5,6,7,8,9,10]. It is possible that systemic or local inflammatory changes related to upper reproductive tract infections [11], such as increased production of inflammatory mediators because of bacterial colonization, influence the development of endometriosis [12]. 

Pelvic inflammatory disease is caused by an infection of the female upper genital tract. It occurs when pathogenic bacteria spread from the vagina to the uterus, Fallopian tubes, and ovaries [13,14]. Disturbance of the genital tract microbiota has been linked to an increased risk of pelvic infections [13,15,16,17]. Under normal circumstances, a healthy microbiota works to stave off infection, defending against invading pathogens or opportunistic resident microbes. In patients with PID, however, the microbial communities of the vagina are altered sufficiently to result in the overgrowth of harmful microorganisms, these pathogens breach the cervical barrier and ascend, causing dysbiosis and infections of the upper reproductive tract [15,17,18,19]. This suggests that an altered upper reproductive tract microbiota could promote the secretion of inflammatory cytokines and chemokines, thereby facilitating the vascularization and implantation of endometrial tissue in other organs. 

The aim of this study was to investigate the possible association between pelvic inflammatory disease and endometriosis. Using data from the National Health Insurance Research Database (NHIRD), we conducted a retrospective cohort study to determine whether patients with pelvic inflammatory disease (PID) have a higher risk of developing endometriosis.

## 2. Methods

The Taiwanese National Health Insurance Program, which was instituted in 1995, provides coverage for over 99 percent of the population (more than 23 million individuals). Insurance claims data from the National Health Insurance Program are an important source of information for medical research.

In this retrospective cohort study, we used claims data from the Longitudinal Health Insurance Database 2000 (LHID 2000), a subset of the National Health Insurance Research Database (NHIRD). The study was approved by the China Medical University Hospital’s institutional review board. The personal identification numbers used to identify individuals were encrypted; the process of obtaining consent from the individuals included in the study was waived. 

**Figure 1:**
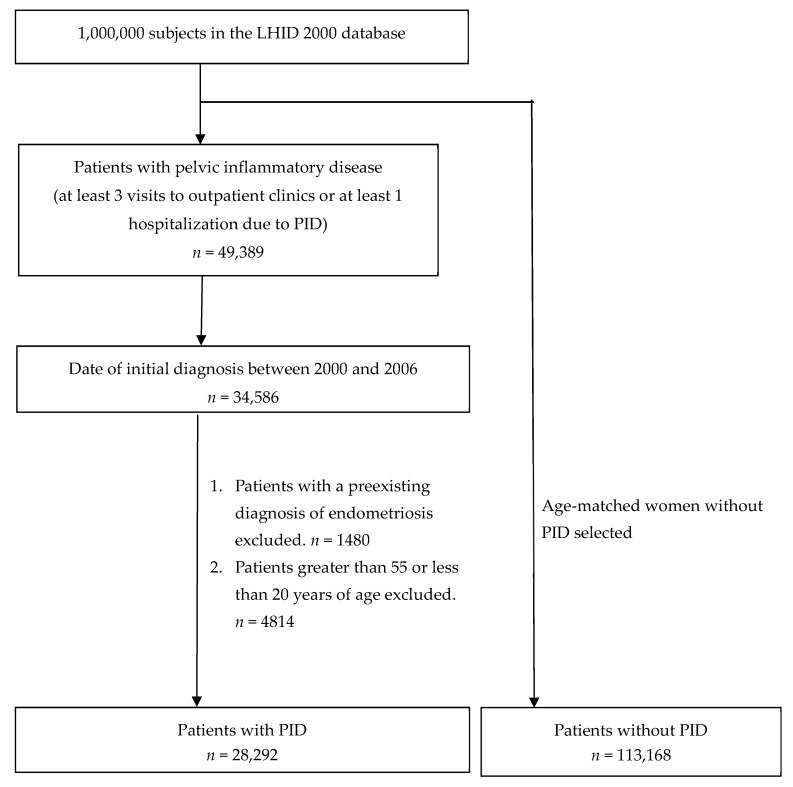
Subject selection process.

The subject selection process is illustrated in Figure 1. After analyzing claims filed by 1,000,000 individuals during the 12-year period from 2000 to 2011, we identified 49,389 individuals who had at least three ambulatory claims or at least one inpatient claim with a diagnosis of pelvic inflammatory disease (ICD-9-CM: 614.3–614.9). Individuals with an initial date of diagnosis during the period from 2000 to 2006 were selected. The initial date of diagnosis was defined as the date of the first outpatient or inpatient visit. Patients with a pre-existing diagnosis of endometriosis were then excluded. Patients greater than 55 or less than 20 years of age were also excluded. A total of 28,292 patients with pelvic inflammatory disease were included in the PID cohort. For each patient with PID, four women in the same five-year age group without an existing diagnosis of PID or endometriosis were randomly selected from the same database. 

The prespecified endpoint of this study was receiving a new clinical diagnosis of endometriosis (ICD-9-CM: 617), with the date of diagnosis being determined by the date of the first outpatient or inpatient claim. A diagnosis of endometriosis was made when endometriotic lesions were identified during laparoscopy or when endometriomas were detected on ultrasound. The follow-up period ended when a diagnosis of endometriosis was established or, in the absence of such a diagnosis, on 31 December 2011. Medical records beyond the end date were not accessed.

Study subjects were divided into the following age groups, 20–29, 30–39, 40–49, and 50–55. Furthermore, to assess the possible impact of pre-existing comorbidities (diagnosed before or at the time of the initial diagnosis of PID) on the development of endometriosis, we identified five associated comorbid conditions: infertility (ICD-9-CM: 628), uterine leiomyoma (ICD-9-CM: 218), autoimmune diseases (including systemic lupus erythematous (ICD-9-CM: 710.0), rheumatoid arthritis (ICD-9-CM: 714.0), and multiple sclerosis (ICD-9-CM: 340)) [20], allergic diseases (asthma (ICD-9-CM: 477), and allergic rhinitis (ICD-9-CM: 477)) [21], and several types of cancer that have been associated with endometriosis (including breast cancer (ICD-9-CM: 174), cervical cancer (ICD-9-CM: 180), and ovarian cancer (ICD-9-CM: 183.0)) [22,23,24,25]. 

The Pearson’s Chi-squared test was used to examine the difference between the expected and observed frequencies and determine the *p*-value for each statistic. A *p*-value of 0.05 was used as the cutoff for statistical significance. We used the Cox proportional hazards model to calculate the hazard ratio (HR) and 95% confidence interval (CI) in patients with PID in comparison to those of controls to assess the risk of developing endometriosis and adjusted for pelvic inflammatory disease, age and comorbidities. The cumulative incidence of endometriosis in the PID and non-case cohorts was calculated with the Kaplan–Meier method; the logrank test was used to test the significance of the difference between the two groups. All datasets were analyzed using with SAS statistical software (version 9.4 for Windows; SAS Institute Inc., Cary, NC, USA). 

## 3. Results

The demographic characteristics and the presence of comorbidities in both cohorts are summarized in Table 1. The infertility rate was significantly elevated in patients with pelvic inflammatory disease. The prevalences of uterine leiomyoma, autoimmune diseases, and allergic diseases were also significantly higher in those with PID. 

The HR and 95% CI for the risk of endometriosis in the disease cohort and the control cohort were analyzed using the Cox proportional hazards regression model. Table 2 shows that the adjusted HRs of endometriosis were significantly higher in patients with pelvic inflammatory disease (HR = 3.02, 95% CI = 2.85–3.2), indicating a higher risk of endometriosis compared to controls. Certain specified comorbidities, including infertility (HR = 1.28, 95% CI = 1.06–1.55), uterine leiomyoma (HR = 2.58, 95% CI = 2.31–2.88), and allergic diseases (HR = 1.24, 95% CI = 1.14–1.35) were also associated with an increased risk of endometriosis. 

Of the 28,292 patients with PID, 2100 (7.42%) were diagnosed with endometriosis during the follow-up period. In contrast, only 2646 (2.33%) out of the 113,168 patients in the control cohort were diagnosed with endometriosis. The rates of newly diagnosed cases of endometriosis were 8.83 and 2.79 per 1000 person-years in the disease and control cohorts, respectively. 

Table 3 shows that after patients with PID were stratified by age and comorbidities, the adjusted HRs for endometriosis were significantly higher in patients of all age groups and in all subgroups stratified by comorbidity except patients with cancer. The highest incidence rate of endometriosis was among patients aged 30–39 both with and without PID. 

Table 4 demonstrates the joint effects of pelvic inflammatory disease and associated comorbidities on the risk of endometriosis. Patients who were diagnosed with pelvic inflammatory disease and infertility (HR = 3.52, 95% CI = 2.72–4.55), uterine leiomyoma (HR = 6.24, 95% CI = 5.35–7.28), autoimmune diseases (HR = 3.80, 95% CI = 2.45–5.91), and allergic diseases (HR = 3.53, 95% CI = 3.12–3.99) had a significantly higher risk of developing endometriosis.

**Figure 2:**
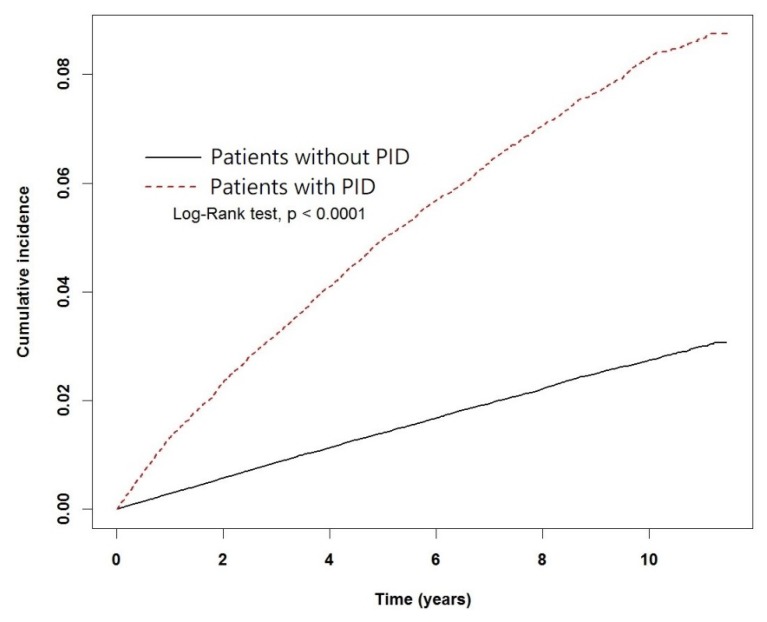
Kaplan-Meier plot of the cumulative incidence of endometriosis in patients with and without PID.

The Kaplan–Meier plot of the cumulative incidence of endometriosis in the PID and control cohorts is shown in Figure 2. The logrank test detected a significant difference between the incidence rates of endometriosis in patients with and without PID.

## 4. Discussion

This nationwide retrospective cohort study involving 141,460 patients demonstrated that patients with PID had a three-fold increase in the risk of developing endometriosis (HR = 3.02, 95% CI = 2.85–3.2) compared with women without PID. Infertility, uterine leiomyoma, and some autoimmune and allergic diseases were also more prevalent in women with PID. It is unknown why the prevalence of cancer was lower in patients with PID.

Though the present study demonstrated a significant association of pelvic inflammatory disease with endometriosis, this association should be interpreted with caution: the correlation between the presence of PID and the development of endometriosis might be due to a causal connection between the two conditions (i.e., that pelvic inflammatory disease somehow predisposes an individual to the development of endometriosis). Or, there could be a confounding third variable—a common pathological process or risk factor that influences the development of both conditions. 

Dysbiosis and inflammation may be the underlying mechanisms linking PID and endometriosis [26]. In 2010, a study showed that higher numbers of the bacteria *Escherichia coli* were present in the menstrual blood of patients with endometriosis. Endotoxin levels in menstrual blood and peritoneal fluid samples were also elevated, most likely due to retrograde menstruation or translocation from the gut [27]. The authors proposed that “bacterial contamination” of the uterine cavity and the peritoneal fluid could promote TLR4-mediated growth of endometrial lesions [28]. The same researchers compared the bacteria present in endometrial swab samples and ovarian cystic fluid (from endometriomas or non-endometriotic cystadenomas) obtained from women with or without endometriosis. Using 16s rRNA sequencing techniques, the study found evidence of subclinical infection in the uterine cavity and also in ovarian endometriomas. Significantly higher numbers of bacteria from the Streptococcaceae and Staphylococcaceae families were present in the cystic fluid of women with ovarian endometriosis [29]. Furthermore, a recent study has shown that certain bacterial markers are associated with an increased risk of developing endometriosis [30]. These findings suggest that the overgrowth of certain bacterial species in the uterine cavity can disrupt the delicate immunological balance of the endometrial microbiota and contribute to the pathogenesis of endometriosis [31]. 

Disequilibrium of the reproductive tract microbiota, such as the abnormal bacterial colonization observed in patients with bacterial vaginosis and PID, could make it easier for pathogenic bacteria to ascend to the upper genital tract [15,17,18]. Studies have shown that pelvic inflammatory disease induces a selective loss of ciliated epithelial cells along the fallopian tube epithelium, which impedes ovum transport, resulting in tubal-factor infertility or ectopic pregnancy [32]. In women with PID, impairment of downward transport could facilitate bacterial contamination of the upper reproductive tract and pelvic cavity, thus predisposing to the development of endometriosis.

Immunologic abnormalities, including chronic inflammation and inadequate immune surveillance in the peritoneum, are also involved in the development of endometriosis [7,11,33,34]. A large body of evidence has associated endometriosis with a higher risk of diseases linked to an altered immune response. These include ovarian and breast cancers, asthma, and other atopic, autoimmune, and cardiovascular diseases [22]. In addition, numerous studies have demonstrated that the production of inflammatory cytokines such as interleukin-1 beta, interleukin-6, and tumor necrosis factor enhances the adhesion of endometrial tissue fragments onto the peritoneum [1,5,6,7,8,9,10]. Though it is unclear whether an altered microbiome is the cause or the effect of impaired host immunity [16], it is logical to assume that, in patients with PID, the presence of pathogenic bacteria in the pelvic cavity could promote the development of endometriosis by causing excessive endometrial inflammation [14]. 

Our study has several limitations. This study was based on a retrospective cohort design, which limited our ability to establish conclusions about causation. Another limitation of this study was the use of insurance claim data, which are dependent on professional ICD coding and are not always complete; this could be a source of misclassification bias in this study. In addition, from the insurance claim data alone, we were unable to determine whether any participants were lost to follow-up because of reasons such as emigration.

It is also important to mention the possibility of ascertainment bias in this analysis: women with PID may have a greater chance of being diagnosed with endometriosis because they are more likely to receive medical investigation of the reproductive tract and pelvic cavity compared to women seeking medical care for non-gynecological conditions. 

Finally, although we adjusted for age and the comorbidities listed above, some confounding variables still remain. Age of menarche, number of births, and use of oral contraceptive pills, body weight, and menstrual cycle length and interval were not analyzed in this study.

In future studies, a prospective cohort design should be used to explore the associations between pelvic inflammatory disease, endometriosis, and other related conditions.

## 5. Conclusions

In conclusion, the results of the present study demonstrate that women with PID are at higher risk of endometriosis compared with women without PID. This suggests that either pelvic inflammatory disease predisposes to endometriosis or that certain mechanisms influence the development of both conditions. Although the underlying processes are not well understood, these findings may have important implications in the diagnosis and management of patients with pelvic inflammatory disease.

## Figures and Tables

**Table 1:** Demographic characteristics and presence of comorbidities in patients with and without pelvic inflammatory disease (PID).

Variables	Pelvic Inflammatory Disease				*p*-Value *
	No (*n* = 113,168)		Yes (*n* = 28,292)		
	*n*	%	*n*	%	
**Age, years (mean ± SD)**	34.50 (9.46)		34.62 (8.91)		
20–29	40,532	35.82	10,133	35.82	
30–39	38,972	34.44	9743	34.44	
40–49	27,868	24.63	6967	24.63	
50–55	5796	5.12	1449	5.12	
**Comorbidity**					
Infertility	1163	1.03	720	2.54	<0.0001
Uterine Leiomyoma	2483	2.19	1442	5.1	<0.0001
Autoimmune diseases	622	0.55	212	0.75	<0.0001
Allergic diseases	11,015	9.73	3694	13.06	<0.0001
Cancer	426	0.38	74	0.26	0.0036
Breast cancer	312	0.28	55	0.19	0.0162
Cervical cancer	106	0.09	19	0.07	0.1795
Ovarian cancer	8	0.01	1	0.00	0.5050
**Follow-up period, years (mean, median)**	8.38 (8.94)		8.41 (8.99)		

* Chi-square test.

**Table 2:** Cox proportional HRs for risk of endometriosis in patients with and without PID, in different age groups, and in patients with preexisting comorbidities.

Characteristics	No. of Patients with Endometriosis	Crude			Adjusted		
	*n* = 4746	HR	(95% CI)	*p*-Value	HR	(95% CI)	*p*-Value
**Pelvic inflammatory disease**							
No	2646	1.00	reference		1.00	reference	
Yes	2100	3.17	(2.99–3.36)	<0.0001	3.02	(2.85–3.2)	<0.0001
**Age, years (mean ± SD)**							
20–29	1334	1.00	reference		1.00	reference	
30–39	2180	1.65	(1.54–1.77)	<0.0001	1.61	(1.5–1.72)	<0.0001
40–49	1185	1.25	(1.15–1.35)	<0.0001	1.14	(1.05–1.24)	0.0012
50–55	47	0.24	(0.18–0.32)	<0.0001	0.22	(0.16–0.29)	<0.0001
**Comorbidity**							
Infertility							
No	4636	1.00	reference		1.00	reference	
Yes	110	1.87	(1.55–2.26)	<0.0001	1.28	(1.06–1.55)	0.0107
Uterine Leiomyoma							
No	4388	1.00	reference		1.00	reference	
Yes	358	3.07	(2.76–3.42)	<0.0001	2.58	(2.31–2.88)	<0.0001
Autoimmune diseases							
No	4711	1.00	reference		1.00	reference	
Yes	35	1.29	(0.93–1.8)	0.1305	1.17	(0.84–1.63)	0.3538
Allergic diseases							
No	4144	1.00	reference		1.00	reference	
Yes	602	1.35	(1.24–1.47)	<0.0001	1.24	(1.14–1.35)	<0.0001
Cancer							
No	4739	1.00	reference		1.00	reference	
Yes	7	0.45	(0.21–0.94)	0.0332	0.54	(0.26–1.14)	0.1058

HR, hazard ratio; CI, confidence interval; Adjusted HR, adjusted for pelvic inflammatory disease, age, and comorbidity in Cox proportional hazards regression.

**Table 3:** Incidence rates, hazard ratios, and confidence intervals of endometriosis in patients with PID stratified by age and comorbidity.

Variables	Pelvic Inflammatory Disease						Crude HR	Adjusted HR
	No (*n* = 113168)			Yes (*n* = 28292)				
	No. of Patients with Endometriosis	Person-Years	IR	No. of Patients with Endometriosis	Person-Years	IR	(95% CI)	(95% CI)
**Total**	2646	948,862	2.79	2100	237,882	8.83	3.17 (2.99–3.36) ***	3.02 (2.85–3.20) ***
**Age, years (mean ± SD)**								
20–29	714	330,919	2.16	620	84,693	7.32	3.39 (3.05–3.78) ***	3.26 (2.93–3.64) ***
30–39	1254	330,618	3.79	926	82,392	11.24	2.96 (2.72–3.23) ***	2.81 (2.58–3.07) ***
40–49	654	238,856	2.74	531	58,540	9.07	3.32 (2.96–3.72) ***	3.1 (2.76–3.48) ***
50–55	24	48,469	0.5	23	12,257	1.88	3.82 (2.16–6.77) ***	3.36 (1.88–6.01) ***
**Comorbidity**								
Infertility								
No	2596	939,708	2.76	2040	232,298	8.78	3.18 (3–3.37) ***	3.04 (2.87–3.22) ***
Yes	50	9154	5.46	60	5584	10.75	1.96 (1.35–2.85) ***	1.91 (1.31–2.81) ***
Uterine Leiomyoma								
No	2467	929,351	2.65	1921	226,941	8.46	3.19 (3.01–3.39) ***	3.14 (2.96–3.34) ***
Yes	179	19,511	9.17	179	10,940	16.36	1.77 (1.44–2.18) ***	1.67 (1.36–2.06) ***
Autoimmune diseases								
No	2631	943,764	2.79	2080	236,230	8.8	3.16 (2.98–3.35) ***	3 (2.83–3.18) ***
Yes	15	5097	2.94	20	1652	12.11	4.1 (2.1–8) ***	4.22 (2.15–8.25) ***
Allergic diseases								
No	2336	862,918	2.71	1808	209,416	8.63	3.19 (3–3.4) ***	3.05 (2.87–3.25) ***
Yes	310	85,943	3.61	292	28,465	10.26	2.84 (2.42–3.34) ***	2.75 (2.34–3.23) ***
Cancer								
No	2640	945,576	2.79	2099	237,278	8.85	3.17 (2.99–3.36) ***	3.02 (2.85–3.2) ***
Yes	6	3285	1.83	1	604	1.66	0.93 (0.11–7.71)	0.82 (0.1-7)

IR, incidence rate, per 1000 person-years; HR, hazard ratio; CI, confidence interval; Adjusted HR, adjusted for pelvic inflammatory disease, age and comorbidity based on Cox proportional hazards regression. *** *p* < 0.001.

**Table 4:** Effects of associated comorbidities on the risk of endometriosis in patients with and without PID.

Variable		Adjusted HR (95% CI)
**Pelvic inflammatory disease**	**Infertility**	
No	No	1 (reference)
No	Yes	1.89 (1.43–2.50) ***
Yes	No	3.05 (2.87–3.23) ***
Yes	Yes	3.52 (2.72–4.55) ***
**Pelvic inflammatory disease**	**Uterine Leiomyoma**	
No	No	1 (reference)
No	Yes	3.56 (3.05–4.16) ***
Yes	No	3.15 (2.96–3.34) ***
Yes	Yes	6.24 (5.35–7.28) ***
**Pelvic inflammatory disease**	**Autoimmune diseases**	
No	No	1 (reference)
No	Yes	1.01 (0.61–1.68)
Yes	No	3.01 (2.84–3.19) ***
Yes	Yes	3.80 (2.45–5.91) ***
**Pelvic inflammatory disease**	**Allergic diseases**	
No	No	1 (reference)
No	Yes	1.29 (1.15–1.45) ***
Yes	No	3.06 (2.87–3.25) ***
Yes	Yes	3.53(3.12–3.99) ***
**Pelvic inflammatory disease**	**Cancer**	
No	No	1 (reference)
No	Yes	0.65 (0.29–1.44)
Yes	No	3.02 (2.85–3.20) ***
Yes	Yes	0.59 (0.08–4.16)

HR, hazard ratio; CI, confidence interval; Adjusted HR: adjusted for pelvic inflammatory disease, age, and comorbidity in Cox proportional hazards regression. *** *p* < 0.001.
